# In Vitro Assessment of the Bioaccessibility and Hypoglycemic Properties of Essential Amino Acids Blend: Implication for Diabetes Management

**DOI:** 10.3390/nu17162606

**Published:** 2025-08-11

**Authors:** Lorenza d’Adduzio, Melissa Fanzaga, Maria Silvia Musco, Marta Sindaco, Paolo D’Incecco, Giovanna Boschin, Carlotta Bollati, Carmen Lammi

**Affiliations:** 1 Department of Pharmaceutical Sciences, University of Milan, Via Mangiagalli, 25, 20133 Milan, Italy; lorenza.dadduzio@unimi.it (L.d.); melissa.fanzaga@unimi.it (M.F.); maria.musco@unimi.it (M.S.M.); giovanna.boschin@unimi.it (G.B.); carlotta.bollati@unimi.it (C.B.); 2 Department of Food, Nutrition and Environmental Sciences, University of Milan, Via Celoria, 2, 20133 Milan, Italy; marta.sindaco@unimi.it (M.S.); paolo.dincecco@unimi.it (P.D.)

**Keywords:** amino acids, Caco-2 cells, diabetes, DPP-IV, GLP-1

## Abstract

**Background/Objectives:** Essential amino acid (EAA) supplementation is often employed in sportive and clinical nutrition due to EAAs’ role in muscle mass maintenance and growth. EAAs are also involved in insulin and glucagone regulation in diabetes management, but only few reports investigate their possible implication as dipeptidyl peptidase-IV (DPP-IV) inhibitors and their effect on the stability and secretion of enteroendocrine hormones. A blend of EAAs (called GAF) available as a food supplement, in a specific qualitative and quantitative ratio, was investigated to address its in vitro bioaccessibility, its hypoglycemic properties in vitro and in situ on cellular models, and its safety on intestinal Caco-2 cells. **Methods**: GAF was subjected to the INFOGEST static digestion protocol, producing the iGAF sample. iGAf DPP-IV inhibitory properties were investigated both in vitro and in situ on Caco-2 cells. Then, STC-1 enteroendocrine cells were employed alone and in co-culture with Caco-2 cells to evaluate iGAF’s impact on glucagon-like peptide 1 (GLP-1) hormone secretion. **Results:** The study demonstrates that the present EAAs blend is stable and bioaccessible after simulated gastrointestinal digestion, and it is safe at the intestinal cellular level. It inhibits DPP-IV enzyme both in vitro and in situ and promotes GLP-1 secretion by enteroendocrine cells. **Conclusions:** The sample demonstrated safety at the intestinal level and showed hypoglycemic properties by acting on a dual synergic mechanism that involves DPP-IV enzyme inhibition and GLP-1 hormone stimulation.

## 1. Introduction

Amino acids (AAs) are small organic molecules that play a central role in biological systems as the structural units of proteins. In humans, twenty different amino acids are involved in protein synthesis, of which some of them are categorized as essential amino acids (EAAs), i.e., isoleucine, leucine, lysine, methionine, phenylalanine, threonine, tryptophan, and valine; they are called essential, since cannot be synthesized de novo by humans or other mammals and must be consumed through dietary intake [1].

The importance of EAAs for human health is well established [2]; they are crucial for maintaining nitrogen balance [3], supporting muscle growth and repair [4], regulating immune responses, producing neurotransmitters (e.g., tryptophan for serotonin, phenylalanine for dopamine) [5,6] and hormones, and enabling numerous metabolic pathways [7]. Inadequate intake of essential amino acids can lead to protein–energy malnutrition, muscle wasting, impaired immunity, delayed wound healing, and developmental disorders, particularly in vulnerable populations such as infants, the elderly, and individuals with chronic illnesses [1,8].

For these reasons, in recent years amino acid supplementation has emerged as a practical strategy to support optimal health, physical performance, and recovery [9,10]. Supplementation with free-form amino acids or balanced mixtures of EAAs is commonly used in clinical nutrition to prevent muscle atrophy in bedridden or elderly patients and to improve outcomes in conditions involving metabolic stress. In sportive nutrition, amino acid supplements, especially those containing branched-chain AAs (BAAs), such as leucine, isoleucine, and valine, are used to enhance muscle protein synthesis, reduce exercise-induced muscle damage, and promote faster recovery [11].

EAA supplementation supports not only muscle health and protein synthesis but also plays a role in glucose metabolism and insulin secretion [12]. It is known that AAs, in particular, leucine, isoleucine, alanine, and arginine, enhance glucose-stimulated insulin secretion from β cells [13]. In greater detail, AAs modulate insulin release through mechanistic targeting of rapamycin (mTORC1), AMP-activated protein kinase (AMPK), and incretin signaling. Together, these mechanisms significantly promote insulin secretion in patients with type 2 diabetes [14]. When combined with glucose, AAs from food proteins, and released by intestinal epithelial cells, promote insulin secretion, which, in turn, promotes protein synthesis and amino acid transport in target tissues like skeletal muscle [15,16]. The metabolic effects vary for each amino acid, with isoleucine and phenylalanine lowering glucose levels; however, leucine significantly enhances insulin production when consumed alongside glucose [17]. In addition to insulin and glucagon stimulation, several glucoregulatory hormones contribute to glucose homeostasis; these include, in particular, the incretins glucagon-like peptide 1 (GLP-1) and glucose-dependent insulinotropic peptide (GIP), two peptide hormones secreted from L- and K-enteroendocrine cells of the intestine, respectively [18]. Both GLP-1 and GIP potentiate glucose-dependent insulin secretion; with GLP-1, this also inhibits glucagon secretion [19]. Interestingly, in addition to their direct insulinotropic effect, AAs may contribute to glucose homeostasis by affecting incretin hormones’ release and stability. Indeed, since GLP-1 is rapidly metabolized by dipeptidyl peptidase-IV (DPP-IV), DPP-IV inhibitors protect GLP-1 degradation with positive outcomes on insulin secretion and glucose homeostasis. As early as 1966, Floyds and colleagues [20] demonstrated that intravenous infusion of a mixture of L-amino acid elevated plasma insulin levels, consequently reducing blood glucose concentrations in healthy individuals; the same researchers noted that a similar response was observed following the infusion of individual amino acids [21]. Nevertheless, other later studies underlined some differences in the capacity of the various AAs to stimulate insulin secretion [22,23]. In this context, the EAA blend (Gunaminoformula, GAF) is available on the market as a dietary supplement containing a mixture of eight EAAs in a specific qualitative and quantitative ratio (L-Leucine, 200 mg/g; L-Valine, 160 mg/g; L-Isoleucine, 150 mg/g; L-Lysine, 140 mg/g; L-Phenylalanine, 130 mg/g; L-Threonine, 110 mg/g; L-Methionine, 70 mg/g; L-Tryptophan, 40 mg/g). It has been formulated to meet the EAA supplementation needs of athletes for muscle recovery and performance, of elderly individuals for the prevention of muscle loss, and for people with restrictive diets (e.g., vegetarians or vegans) to ensure adequate amino acid intake.

Therefore, with the goal of advancing and precisely characterizing the health promoting ability of GAF, this study adopts a comprehensive multidisciplinary approach to deeply investigate GAF’s biological properties in vitro. It uncovers novel targets and elucidates previously unrecognized mechanism of action, shedding light on how GAF may exert its unique hypoglycemic potential.

In greater detail, firstly, GAF was subjected to the well-known INFOGEST static digestion protocol to assess its bioaccessibility (obtaining the final iGAF sample). Thus, iGAF’s ability to inhibit DPP-IV enzyme both in vitro and in situ on Caco-2 cells was evaluated. Moreover, iGAF’s impact on GLP-1 hormone secretion and stability was investigated in STC-1 enteroendocrine cells and in a co-culture system of Caco-2 and STC-1 cells. Particularly, the co-culture model was established to simulate the intestinal barrier environment in which GLP-1 is physiologically produced by L cells; for this purpose, STC-1 cells were used as a validated murine enteroendocrine model capable of secreting GLP-1 upon stimulation [24]. Lastly, iGAF safety at the intestinal level was assessed.

## 2. Materials and Methods

### 2.1. Chemicals

Gunaminoformula sample was produced by Guna S.p.a (Milano, Italy). The complete list of chemicals is available in Supplementary Materials.

### 2.2. GAF Static In Vitro Digestion with INFOGEST Protocol

Following the recommended daily dose of the producer, five GAF tablets (5 g) were mechanically pulverized in a mortar. Table S1 reports that 5 GAF tablets contain 1 g of L-leucine, 0.8 g of L-Valine, 0.75 g of L-Isoleucine, 0.7 g of L-Lysine, 0.65 g of L-Phenylalanine, 0.55 g of L-Threonine, 0.35 g of L-Methionine, and 0.2 g of L-Tryptophan. The in vitro static digestion was carried out in accordance with previously described INFOGEST protocol [25]. More detailed information is available in Supplementary Materials.

### 2.3. Amino Acid (a.a.) Analysis

The GAF and iGAF samples were diluted 1:1 with 0.2 N lithium citrate buffer pH 2.2. Details are provided in Supplementary Materials section.

### 2.4. Cell Culture

Caco-2 cells and STC-1 cells were routinely sub-cultured following a previously optimized protocol [24]. For the co-culture, the STC- 1 and Caco-2 cells were cultured in a 1:5 ratio for 48 h before proceeding with treatments. Details are provided in Supplementary Materials.

### 2.5. Caco-2 Cell Differentiation

For Caco-2 cell differentiation, cells were seeded onto polycarbonate Transwell filters as previously described [26]. Supplementary Materials contain further explanations.

### 2.6. Caco-2 Cells Monolayers Integrity Evaluation

The transepithelial electrical resistance (TEER) of differentiated Caco-2 cells was measured at 37 °C immediately before and after 15, 30, 60, and 120 min. More detailed information is available in Supplementary Materials.

### 2.7. Amino Acid Uptake by Caco-2 Cell Monolayers

iGAF intestinal absorption was assessed in transport buffer solution (137 mM of NaCl, 5.36 mM of KCl, 1.26 mM of CaCl_2_, 1.1 mM of MgCl_2_, and 5.5 mM of glucose). The apical solutions were kept at pH 6.0 (buffered with 10 mM of morpholinoethane sulfonic acid) and the basolateral (BL) solutions were kept at pH 7.4 (buffered with 10 mM of N2hydroxyethylpiperazine-N4butanesulfonic acid) to replicate the pH conditions found in vivo in the small intestinal mucosa. Full experimental condition is reported in the Supplementary Materials.

### 2.8. Cell Treatment Conditions

The experiments at cellular level on Caco-2 cells were designed to closely mimic the physiological conditions of intestinal absorption of the digested product, composed of amino acids. Specifically, since the recommended GAF daily dose is 5 tablets (5 g of product), and since the final yield after simulated digestion using the INFOGEST protocol is 3.6 g of dry product, it is assumed that this amount of digested material would theoretically interact with an absorptive intestinal surface. In particular, the surface area of the duodenal intestinal tract was calculated following the previously described formula [27]. More detailed information is available in the Supplementary Materials section.

### 2.9. 3-(4,5-Dimethylthiazol-2-yl)-2,5-Diphenyltetrazolium Bromide (MTT) Assay

A total of 3 × 10^4^ Caco-2 cells/well and 6 × 10^3^ STC-1 cells/well were seeded in 96-well plates. Caco-2 cells were treated with GAF, iGAF, and IB (10 mg/cm^2^) or vehicle (H_2_O) in complete growth medium for 2 h at 37 °C under a 5% CO_2_ atmosphere, following the procedure previously reported [28]. STC-1 cells were treated with iGAF up to 10 mg/cm^2^. For the co-culture system, a total of 2.4 × 10^4^ Caco- 2 cells and 6 × 10^3^ STC-1 cells/well were seeded in 96-well plates and treated with iGAF and/or vehicle (H_2_O) at 0.3 and 1.5 mg/cm^2^, following the same conditions described above. In 96-well plates with a surface area of 0.32 cm^2^, the cells were treated with 10 mg/cm^2^ of sample and received 3 mg of sample.

### 2.10. Antidiabetic Activity of GAF

#### 2.10.1. In Vitro Measurement of the DPP-IV Inhibitory Activity

The experiments were carried out in triplicate in a half-volume, 96-well solid plate (white) using previously optimized conditions [29]. More detailed information is available in the Supplementary Materials section.

#### 2.10.2. Evaluation of the Inhibitory Effect of iGAF on Cellular DPP-IV Activity

A total of 3 × 10^4^ Caco-2 cells/well were seeded in black 96-well plates with clear bottom. Details are provided in Supplementary Materials section.

#### 2.10.3. Evaluation of the GLP-1 Stability and Secretion at Cellular Level

STC-1 GLP-1 secretion was measured with an active GLP-1 ELISA kit (catalog no. EGLP-35K; Millipore, Watford, UK). Details are provided in Supplementary Materials section.

### 2.11. Statical Analysis

Results were presented as mean ± standard deviation (s.d.), and all measurements were carried out at least in triplicate. All the data sets were checked for normal distribution by D’Agostino and Pearson testing. Since they were all normally disturbed with *p*-values < 0.05, we proceeded with statistical analyses with *p*-values < 0.05 deemed significant. Dunnett’s and Tukey’s post-tests were conducted after statistical analyses using one- and two-way ANOVA (Graphpad Prism 9, GraphPad Software 9, La Jolla, CA, USA).

## 3. Results

### 3.1. GAF and iGAF Amino Acid Composition Analysis

According to the nutritional label (Table S1), GAF contains 20% L-Leu (200 mg/g), 16% L-Val (160 mg/g), 15% L-Ile (150 mg/g), 14% L-Lys (140 mg/g), 13% L-Phe (130 mg/g), 11% L-Thr (110 mg/g), 7% L-Met (70 mg/g), and 4% of L-Trp (40 mg/g). For the bioaccessibility study, 5 g of GAF was ground, dissolved in 5 mL of water, and subjected to the in vitro static INFOGEST protocol. Gastric digestion was performed with simulated gastric fluid containing pepsin, followed by intestinal digestion with simulated intestinal fluid (1:1 *vol*/*vol*) containing pancreatin and bile salts. The enzymes were inactivated at 95 °C for 10 min. The IB was prepared using water instead of GAF. The final INFOGEST product was centrifugated to remove the insoluble digested fraction, and the supernatant containing the soluble fraction underwent a molecular weight cut-off (3 kDa cut-off) to remove the already thermically inactivated enzymes of the digestive phases and concentrate the bioaccessible AAs recovered in the filtrate solution.

**Figure 1:**
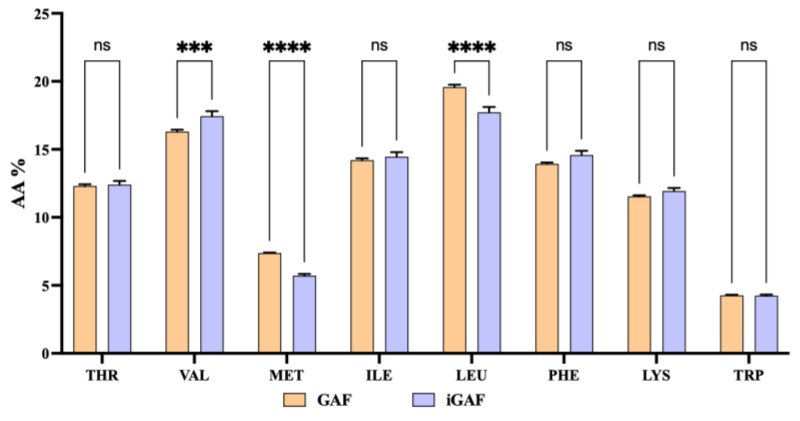
Amino acid distribution (%) in Gunaminoformula (GAF) and INFOGEST Gunaminoformula (iGAF) samples. The data points represent the averages ± SD of 4 independent experiments (technical replicates) performed in triplicate (biological replicates). All data sets were analyzed by one-way ANOVA followed by Tukey’s post hoc test. ns: not significant; (***) *p* ≤ 0.001; (****) *p* ≤ 0.0001. THR: threonine; VAL: valine; MET: methionine; ILE: isoleucine; LEU: leucine; PHE: phenylalanine; LYS: lysine; TRP: tryptophan.

A parallel analysis was performed on GAF and iGAF to evaluate whether the INFOGEST protocol modified the AA percentage in the final sample by impacting on AA bioaccessibility. The results in Figure 1 demonstrate that the amino acid profile, expressed as percentage composition, was consistent between GAF and iGAF for almost all amino acids. This result indicates that the gastrointestinal digestion did not modify the proportional representation of amino acids during digestion. Indeed, no significant loss or alteration in amino acid percentage was observed post-digestion, except for L-Met and L-Leu, for which the INFOGEST protocol leads to a slight but significant percentage reduction, and for L-Val, whose percentage was significantly increased in the INFOGEST sample. The differences in amino acid concentrations reflect changes in their bioaccessibility. In greater detail, the observed variations in some amino acid levels may result from interactions with residual tablet excipients (e.g., cellulose) or differences in solubility and release during digestion, mimicked using the INFOGEST protocol. These matrix-related effects can influence how single amino acids become available in the soluble fraction after digestion.

### 3.2. Ability of GAF and iGAF to Inhibit in Vitro DPP-IV Enzyme Activity

**Figure 2:**
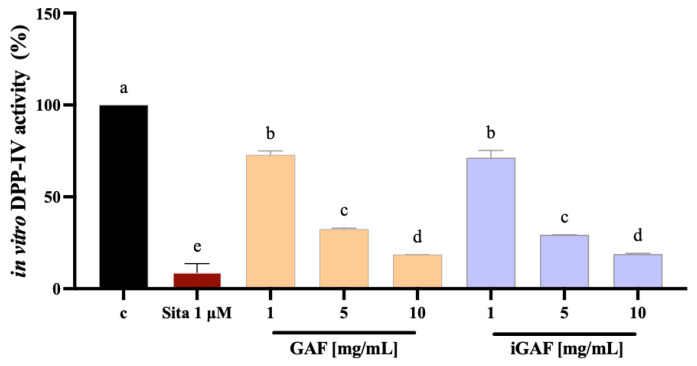
Effect of Gunaminoformula (GAF) and INFOGEST Gunaminoformula (iGAF) on the in vitro DPP-IV activity. The data points represent the averages ± SD of 4 independent experiments (technical replicates) performed in triplicate (biological replicates). All data sets were analyzed by one-way ANOVA followed by Tukey’s post hoc test. ns: not significant. Different lowercase letters indicate a significant difference (*p* < 0.05) between different treatments. C: control sample (H_2_O). Sita: sitagliptin, 1 μM.

To evaluate GAF’s antidiabetic potential, dedicated experiments were performed to investigate its ability to modulate in vitro DPP-IV activity. In greater detail, experiments were carried out assessing both the original sample of GAF and iGAF. As reported in Figure 2, in a cell-free in vitro assay, both the samples reduced DPP-IV activity in a comparable and dose-dependent manner relative to the control group (C). Specifically, at 1.0, 5.0, and 10.0 mg/mL, GAF was able to bring the DPP-IV enzyme activity up to 72.76 ± 2.248%, 32.41 ± 0.5768%, and 18.56 ± 0.1466%, respectively. At the same concentrations, iGAF was able to suppress DPP-IV activity by 28.68 ± 4.01%, 70.68 ± 0.02%, and 81.13 ± 0.35%, respectively. Sitagliptin, the internal positive control, reduced DPP-IV activity by 91.19 ± 4.84%. On the contrary, the IB sample did not exhibit inhibitory activity on DPP-IV enzyme (Figure S1).

### 3.3. iGAF’s Ability to Inhibit in Situ DPP-IV Enzyme Activity on Human Intestinal Caco-2 Cells

Previous results clearly suggested that simulated gastrointestinal digestion (by INFOGEST) did not impact either the GAF AA composition nor its capacity to in vitro inhibit the DPP-IV activity. In light of these results, we can claim that GAF AA is fully bioaccessible, and therefore, considering that from a physiological point of view, the GAF original sample would not reach the intestinal cells as it is, further investigations on intestinal cells were only focused on and carried out on the iGAF sample.

**Figure 3:**
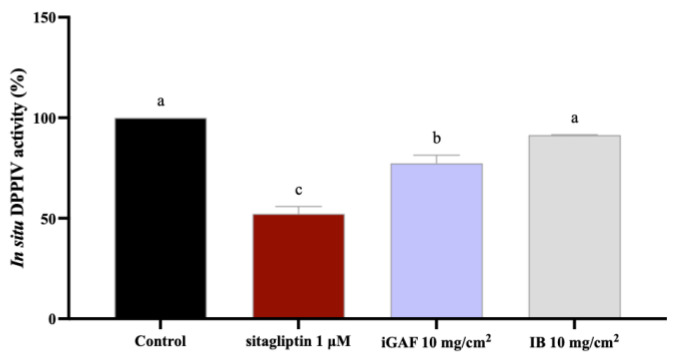
Effect of INFOGEST Gunaminoformula (iGAF) on the cellular DPP-IV activity. The data points represent the average ± SD of 4 independent experiments (technical replicates) performed in triplicate (biological replicates). All data sets were analyzed by one-way ANOVA followed by Tukey’s post hoc test. ns: not significant. Different lowercase letters indicate a significant difference (*p* < 0.05) between different treatments. IB: INFOGEST blank. Control: control sample (H_2_O).

Indeed, given the promising results obtained on the purified enzyme, iGAF was subsequently tested on the human intestinal Caco-2 cell line, the cells of which express DPP-IV enzyme on their surface membrane [30], to evaluate and confirm its ability to reduce enzymatic activity in a more realistic cellular environment. Cell-based experiments on Caco-2 cells were designed to replicate in vitro the intestinal sample-to-surface ratio of iGAF. Starting from 5 g of GAF, the INFOGEST protocol yielded 3.6 g of dried iGAF. This iGAF amount would physiologically encounter a duodenum absorptive area of ~2450 cm^2^, estimated using the formula previously described in the Materials and Methods section. To replicate, at the cellular level, this sample-to-surface ratio, 3 mg of iGAF was applied to each well (0.32 cm^2^ × 6.5 = ~2 cm^2^ effective surface area), corresponding to a dose of 10 mg/cm^2^. In 96-well plates, each well received 3 mg of iGAF. MTT experiments excluded any possible cytotoxic effect of iGAF (10 mg/cm^2^) on Caco-2 cells, as reported in Figure S2; thus, of GAF (10 mg/cm^2^, corresponding to 3 mg/well) was assayed on intestinal cells to screen its potential to inhibit transmembrane DPP-IV enzyme activity. Data reported in Figure 3 show that after 15 min of treatment, iGAF inhibits in situ the cellular DPP-IV enzyme by 22.65 ± 4.02% compared to control (C) conditions, while the positive control sitagliptin was able to inhibit cellular DPP-IV activity by 47.82 ± 3.71% at the final tested concentration of 1 μM. In accordance with the in vitro DPP-IV activity results, the IB sample did not show any in situ DPP-IV inhibitory properties.

### 3.4. iGAF’s Ability to Induce GLP-1 Hormone Secretion in Enteroendocrine STC-1 Cells and to Modulate Its Stability in the STC-1/Caco-2 Co-Culture System

To assess the impact of potential DPP-IV inhibitors on GLP-1 level modulation, a co-culture model of Caco-2 and STC-1 cells was developed to evaluate both hormone stability and secretion levels, respectively [24].

**Figure 4:**
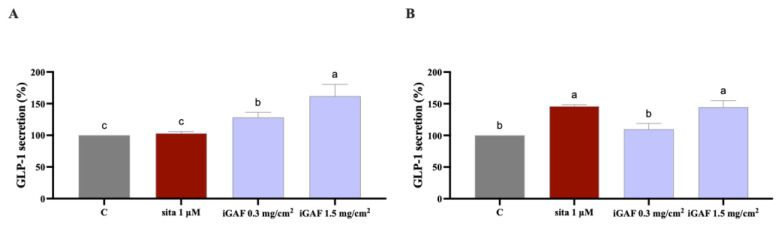
INFOGEST Gunaminoformula (iGAF) effect on GLP-1 secretion. (**A**) Effect of iGAF on GLP-1 secretion in STC-1 cell line. (**B**) Effect of iGAF on GLP-1 in co-culture Caco-2/STC-1 cells. Data points represent averages ± s.d. of three independent experiments (technical replicates) performed in triplicate (biological replicates). All data sets were analyzed by two-way ANOVA followed by Tukey’s post hoc test. ns: not significant. Different lowercase letters indicate a significant difference (*p* < 0.05) between different treatments. C: control sample. Sita: sitagliptin.

Firstly, results suggested that iGAF overall did not exhibit a cytotoxic effect on STC-1 (Figure S3); hence, 0.3 and 1.5 mg/cm^2^ of iGAF (corresponding to 1 and 5 mg/mL, respectively) were selected for subsequent experiments aimed at evaluating its ability to stimulate GLP-1 secretion in STC1 cells in comparison to the untreated control basal condition. Data reported in Figure 4A suggest that the sample significantly increases the concentration of GLP-1 in cell culture media after 1 h treatment up to 128.30 ± 7.91% and 161.80 ± 18.59% at the final assayed amounts of 0.3 and 1.5 mg/cm^2^, respectively. Sitagliptin (1 µM) did not significantly increase GLP-1 secretion in STC-1 cells, bringing GLP-1 values up to 103.3 ± 2.608%.

To further investigate its biological activity under conditions that better mimic the intestinal environment, the sample was subsequently tested in a co-culture system of Caco-2 enterocytes and STC-1 enteroendocrine cells with the aim of evaluating its potential to enhance the stability of the GLP-1 hormone at the cellular level. Data reported in Figure 4B shows that in the co-culture system, treatment with iGAF (1.5 mg/cm^2^) resulted in a 144.60 ± 10.27% increase in GLP-1 secretion compared to the untreated control. A comparable effect was observed with sitagliptin (1 μM), which induced a 146.10 ± 2.29% increase in GLP-1 production. In contrast, iGAF treatment at 0.3 mg/cm^2^ led to a GLP-1 secretion level similar to the control (109.80 ± 9.05%).

### 3.5. TEER Measurements in Differentiated Caco-2 Cells

**Figure 5:**
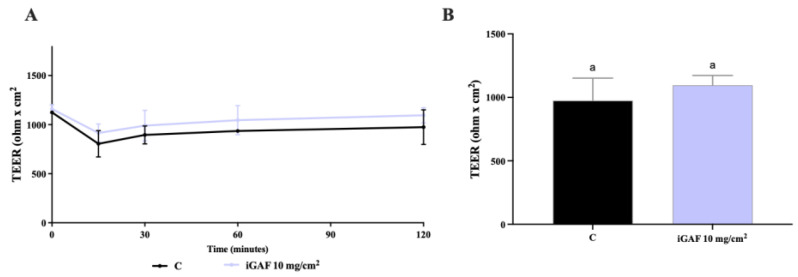
Transepithelial electrical resistance (TEER) measurements in differentiated Caco-2 monolayer. (**A**) Time course of TEER changes recorded in 2 h in untreated (control) and GAF-treated Caco-2 cells. The same “a” lowercase letters indicate that they are not significant difference (*p* < 0.05) between different treatments. (**B**) TEER values after 120 min. Data are the mean ± S.D. of experiments performed in triplicate (biological replicates). C: untreated cells. iGAF: INFOGEST Gunaminoformula.

To evaluate the integrity of the differentiated Caco-2 cells’ epithelial monolayer when in contact with iGAF tested at 10 mg/cm^2^, TEER values were measured. The results shown in Figure 5A,B indicate that iGAF did not alter the intestinal monolayer permeability. These results, in line with MTT assay results on Caco-2 cells, clearly show that GAF is safe on Caco-2 cells and does not demonstrate negative effects on intestinal cells’ permeability.

## 4. Discussion

Our study provides new evidence regarding the antidiabetic activity of GAF AA formulation, unlocking a new mechanism of action which is linked to the direct ability of this product to modulate DPP-IV activity, leading to an improvement in GLP-1 stability and cellular production and secretion. Overall, our results are in line with previous evidence suggesting that AAs can influence glucose homeostasis by direct and indirect insulinotropic mechanisms. For instance, cationic amino acids, such as lysine and arginine, act directly by enhancing β-cell depolarization; this leads to calcium channel activation, with intracellular calcium influx, ultimately resulting in insulin exocytosis [31,32]; other direct mechanisms involve mTORC1 activation in β cells. AAs can also impact on glucose metabolism, with indirect insulinotropic mechanisms, by acting on the enteroendocrine system and parasympathetic nervous system. It is well known that the ingestion of dietary proteins and amino acids stimulates the secretion of GLP-1, a gut hormone that suppresses appetite and lowers blood sugar [16,33]. Interestingly, it has been shown that a number of amino acids are strong inducers of GLP-1 release both in vivo and in vitro [34,35]. Notably, our findings suggest that the qualitative and quantitative EAA formulation contained in GAF may be directly involved in regulating glucose homeostasis through its direct ability to modulate DPP-IV activity. Indeed, firstly it was demonstrated—through the application of INFOGEST, an in vitro static digestion protocol that is widely employed, since it has been shown to be very useful in predicting outcomes of in vivo digestion—that GAF is stable in simulated gastrointestinal digestion, confirming that EAAs in the iGAF product are completely bioaccessible (Figure 1). Hence, once iGAF was tested in vitro on the DPP-IV target, it directly reduced the target activity with a dose–response trend and with a potency which was comparable to the original GAF product (the one which was not subjected to the INFOGEST protocol, Figure 2). This result suggests that GAF bioaccessibility is not impacted by gastrointestinal digestion. Moreover, these results are, overall, in line with previous studies indicating that Thr, Leu, and Met AAs are competitive inhibitors of DPP-IV enzyme in vitro [36,37]. Other authors identified His, Leu, Ile, and Met as the most potent in vitro DPP-IV inhibitors in a low-molecular-mass extract containing free AAs [37]. In light of this evidence, it appears clear that GAF contains all the above-mentioned AAs; this can explain its DPP-IV inhibition ability, which was also confirmed at the cellular level. Indeed, for the first time, this study demonstrates that bioaccessible GAF modulates DPP-IV activity expressed by human intestinal Caco-2 cells. The human intestinal Caco-2 cell line exhibits numerous morphological and functional features characteristic of enterocytes and naturally expresses a broad array of membrane-bound peptidases on the apical surface of its cells, including DPP-IV. Aiming to replicate as closely as possible the physiological conditions of contact between the sample and intestinal cells, before conducting these experiments, several factors were considered when designing the study at the cellular level. Thus, for MTT experiments, Caco-2 cells were treated with iGAF at 10 mg/cm^2^, and the results (Figure S2) reveal that GAF does not negatively impact on Caco-2 cells’ viability. Interestingly, the cell-based DPP-IV activity assay (Figure 3) revealed that the 15 min pre-incubation with GAF (10 mg/cm^2^) inhibits DPP-IV enzyme expressed on Caco-cells. The intestinal epithelial cell layer is the first significant barrier to absorption that AAs face from a physiological perspective. Assessment of the current AA mixture’s DPP-IV inhibitory activity on intestinal cells offers additional benefits over the in vitro assay using the purified enzyme because it replicates the intestinal environment and its transport and metabolic processes [38]. These results are particularly interesting. It is known that the proteolytic activity of the DPP-IV enzyme causes less than 25% of GLP-1 newly secreted by enteroendocrine cells to leave the gut in an intact, active state [39,40]. Furthermore, only 10–15% of freshly released GLP-1 enters the systemic circulation in its intact form, since 40–50% of GLP-1 degradation occurs later in the liver. Additionally, considering that the DPP-IV enzyme is widely expressed and that it is present at the intestinal level in both the endothelial cells lining the capillaries of the lamina propria and the enterocyte brush border, DPP-IV inhibition may be crucial in preventing GLP-1 breakdown.

The apical surface of the L-cell, an open-type intestinal epithelial endocrine cell, has direct contact with luminal nutrients, while the basolateral surface comes into contact with neural and vascular tissue. Numerous dietary, neurological, and endocrine factors permit intestinal L-cells to secrete GLP-1. The main physiological stimulus for GLP-1 release is meal consumption, especially when it involves foods high in fats and carbohydrates [41]. Considering this and given GAF’s ability to inhibit cellular DPP-IV, it is important to highlight that our findings demonstrate GAF’s potential to enhance the GLP-1 via STC-1 cells. Indeed, STC-1 cells are considered a reliable and reproducible enteroendocrine cell model to evaluate secretion of several hormones in response to nutrient components in vitro [42]. In response to various physiological stimuli, STC-1 cells express and secrete a variety of gut hormones that are known to play roles in metabolism, feeding, and satiety. Notably, iGAF INFOGEST stimulates GLP-1 secretion in a dose-dependent manner (Figure 4A). Later, by developing a co-culture system in which both Caco-2 and STC-1 cellular systems were combined, it was possible to dynamically and directly assess the effect on in vitro DPP-IV inhibition that iGAF exerted on GLP-1 production and stability. The experiments were performed using sitagliptin (1 μM) as a reference compound that inhibits DPP-IV enzyme. Our findings indicate that iGAF, when tested at 1.5 mg/cm^2^, can stabilize the GLP-1 hormone present in the cell culture medium of the co-culture model in a manner comparable to the positive control sitagliptin (1 µM).

The results in Figure 4B demonstrate that iGAF works dynamically on two levels, by inhibiting DPP-IV and preventing GLP-1 degradation, and in parallel, stimulating GLP-1 secretion by STC-1 cells. It is important to clarify that GLP-1 stimulation is distinct from GLP-1 secretion. Specifically, GLP-1 stimulation was measured in STC-1 cells alone (Figure 4A). When using the co-culture system, we assessed DPP-IV inhibition and the dynamic stability of GLP-1 (attributed to the presence of Caco-2 cells), as well as GLP-1 secretion (due to the concurrent presence of STC-1 cells). The observation that, at the lowest dose, GAF does not stimulate GLP-1 secretion but still maintains GLP-1 stability in the co-culture system can be explained by an important functional feature of this system: Caco-2 cells exhibit an absorptive phenotype and actively uptake amino acids from the extracellular environment, reflecting the physiological behavior of intestinal epithelial cells (Figure S4). Therefore, since Caco-2 cells potentially uptake some amino acids, as also indicated in Figure S4, the mixture of essential amino acids (at the lowest dose) is unable to stimulate GLP-1 secretion from STC-1 cells; it is only able to maintain its stability. Our explanation is clearly supported by the behavior of sitagliptin. Indeed, sitagliptin, the reference compound, is ineffective in improving GLP-1 secretion in STC-1 cells alone, but it stabilizes GLP-1 in the co-culture system due to the inhibition of DPP-IV. Hence, considering our results, it appears clear that the hypoglycemic activity in vitro of EAAs is not only exerted by DPP-IV inhibition but also through a potential additional mechanism which led to the GLP-1 stimulation; this mechanism needs to be explored.

These in vitro results clearly show that the present EAA mixture impacts on glucose metabolism, perhaps due to its qualitative and quantitative AA composition. In the literature, several AAs have been demonstrated to stimulate GLP-1 release in vitro and in vivo [43,44,45]. Interestingly, iGAF contains BCAAs (in particular L-Val (17.7%)), which, in previous research, have been demonstrated to be a potent stimulus for GLP-1 secretion when luminally infused in rats, and Leu and Ile (18 and 14.7%, respectively), which, in another study, have been demonstrated to stimulate GLP-1 secretion in a dose-dependent manner in vitro [46]. Interestingly, Raimer and colleagues demonstrated that in response to 2% leucine and isoleucine, there was a 4.7 and 2.6-fold increase in GLP-1 release, respectively. Lastly, according to our results (Figure 5A,B), iGAF (10 mg/cm^2^) did not negatively impact on the TEER values, demonstrating that the present in vitro, digested EAA formulation did not alter the intestinal monolayer permeability. These results are corroborated by the MTT cellular viability assay (Figure S2).

## 5. Conclusions

In conclusion, the present study provides new evidence that GAF is safe at an intestinal level and can positively modulate incretin dynamics in vitro, showing an inhibitory effect on DPP-IV enzyme (with a positive effect on in vitro GLP-1 degradation) and stimulating GLP-1 secretion at the cellular level. These dual actions might contribute to improved glucose regulation and highlight the metabolic potential of EAA supplementation. Our findings, which emphasize the antidiabetic efficacy of EAAs, might be confirmed by also in vivo studies.

## Data Availability

Data is contained within the article and supplementary material: The original contributions presented in this study are included in the article and supplementary materials.

## Supplementary Materials

The following supporting information can be downloaded at: https://www.mdpi.com/article/10.3390/nu17162606/s1, Supplementary Materials and Methods; Figure S1: Effect of INFOGEST blank (IB) on in vitro DPP-IV activity; Figure S2. Evaluation of GAF, iGAF, and IB effect on human intestinal Caco-2 viability; Figure S3. iGAF’s cytotoxicity evaluation on enteroendocrine STC-1 cells’ viability; Figure S4. % of trans-epithelial transported amino acid after INFOGEST Gunaminoformula (iGAF) treatment. Table S1. Gunaminoformula nutritional composition.

## Abbreviations

The following abbreviations are used in this manuscript:

AAs	Amino acids
AMPK	AMP-activated protein kinase
AP	Apical chamber
BCAAs	Branched amino acids
BL	Basolateral chamber
DPP-IV	Dipeptidyl peptidase-IV
EAAs	Essential amino acids
FBS	Fetal bovine serum
GAF	Gunaminoformula
GIP	Gastric inhibitory polypeptide
GLP-1	Glucagon-like peptide-1
IB	INFOGEST blank
iGAF	INFOGEST Gunaminoformula
L-Ile	L-Isoleucine
L-Leu	L-Leucine
L-Lys	L-Lysine
L-Met	L-Methionine
L-Phe	L-Phenylalanine
L-Thr	L-Threonine
L-Trp	L-Tryptophan
L-Val	L-Valin
mTORC1	Mammalian target of rapamycin complex 1
MTT	3-[4,5-dimethylthiazol-2-yl]-2,5 diphenyl tetrazolium bromide
TEER	Transepithelial electrical resistance
SGF	Simulated gastric fluid
SIF	Simulated intestinal fluid
