# Supplementary material for: In Vitro Assessment of the Bioaccessibility and Hypoglycemic Properties of Essential Amino Acids Blend: Implication for Diabetes Management
Source: Nutrients. 2025 Aug 11;17(16):2606. doi: 10.3390/nu17162606 (PMC12389530; doi:10.3390/nu17162606)
Supplement: Supplementary file 1 [file nutrients-17-02606-s001.zip › nutrients-3762886-supplementary.pdf]

nutrients

MDPI

Supplementary information

In vitro Assessment of the Bioaccessibility and Hypoglycemic Properties of Essential Amino Acids Blend: Implication for Diabetes Management

Lorenza d'Adduzio[1], Melissa Fanzaga[1], Maria Silvia Musco[1], Marta Sindaco[2], Paolo D'Incecco[2], Giovanna Boschin[1], Carlotta Bollati[1], Carmen Lammi[1*]

1 Department of Pharmaceutical Sciences, University of Milan, Via Mangiagalli, 25, 20133 Milan, Italy
2 Department of Food, Nutrition and Environmental Sciences, University of Milan, Via Celoria, 2, 20133 Milan, Italy
* Correspondence: carmen.lammi@unimi.it; Tel.: 02503 19372

Academic Editor: Firstname Lastname

Received: date
Revised: date
Accepted: date
Published: date

Citation: To be added by editorial staff during production.

Copyright: © 2025 by the authors. Submitted for possible open access publication under the terms and conditions of the Creative Commons Attribution (CC BY) license (https://creativecommons.org/licenses/by/4.0/).

Table S1. Gunaminoformula nutritional composition.

Nutritional Information	per 100 g	per 5 tablets
Energy	1676 kJ / 395 kcal	85 kJ / 20 kcal
Fats	0.07 g	0 g
of which saturated fatty acids	0 g	0 g
Carbohydrates	0 g	0 g
of which sugars	0 g	0 g
Proteins	0 g	0 g
Salt	0.01 g	0 g
L-Leucine	19.70 g	1000 mg
L-Valine	15.76 g	800 mg
L-Isoleucine	14.78 g	750 mg
L-Lysine	13.79 g	700 mg
L-Phenylalanine	12.81 g	650 mg
L-Threonine	10.84 g	550 mg
L-Methionine	6.90 g	350 mg
L-Tryptophan	3.94 g	200 mg

S.2. Supplementary material and methods

S.2.1. Chemicals

The DPP-IV enzyme and the substrate solution [5 mM H- Gly-Pro conjugated to aminomethylcoumarin (H-Gly-Pro-AMC)] were provided by Cayman Chemicals (Michigan, USA). Porcine pepsin (Sigma-Aldrich, cat. no. P7012), Bovine bile (Sigma-Aldrich, cat. no. B3883), Porcine pancreatin (Sigma-Aldrich, cat. no. P7545), KCl (Merck, cat. no. 4936), KH_2PO_4 (Sigma-Aldrich, cat. no. P0662), $NaHCO_3$ (Merck, cat. no. 6329), NaCl (Merck, cat. no. 6404), $MgCl_2(H_2O)_6$ (Merck, cat. no. 5833), $(NH_4)_2CO_3$ (Sigma-Aldrich, cat. no. 207861), $CaCl_2$ $(H_2O)_2$ (Sigma-Aldrich, cat. no. C3881) were purchased from Sigma Aldrich. (St. Louis, MO, USA). Dulbecco's modified Eagle medium (DMEM), fetal bovine serum (FBS), L- glutamine, phosphate buffered saline (PBS), penicillin/streptomycin, 24 and 96- well plates were from Euroclone (Milan, Italy). Caco-2 cells and STC-1 bought from ATCC (HTB-37 and HB- 8065, respectively, ATCC from LGC Standards, Milan, Italy). Gunaminoformula sample was produced by Guna S.p.a.

S.2.2. GAF static in vitro digestion with INFOGEST protocol.

According to the recommended daily dose of the producer, five GAF tablets (5 g) were mechanically pulverized in a mortar. Table S1 reports that 5 GAF tablets contain 1 g of L-leucine, 0.8 g of L-Valine, 0.75 g of L-Isoleucine, 0.7 g of L-Lysine, 0.65 g of L-Phenylalanine, 0.55 g of L-Threonine, 0.35 g of L-Methionine and 0.2 g of L-Tryptophan. The *in vitro* static digestion was carried out in accordance with previously described INFOGEST protocol (1). For the simulated digestion fluids used in this study (final working volume of 400 mL at 1.25× concentration) electrolyte stock solutions were combined and diluted with water. The electrolyte composition for simulated gastric fluid (SGF) fluid was: KCl 6.9 mM; KH_2PO_4 0.9 mM; $NaHCO_3$ 25 mM; NaCl 47.2 mM; $MgCl_2{\cdot}6H_2O$ 0.12 mM; $(NH_4)_2CO_3$ 0.5 mM; $CaCl_2{\cdot}2H_2O$ 0.15 mM. The pH of the gastric fluid was adjusted to 3.0 using 1 M HCl and monitored using a calibrated pH meter. The electrolyte composition for simulated intestinal fluid (SIF) was: KCl 6.8 mM; KH_2PO_4 0.8 mM; $NaHCO_3$ 85 mM; NaCl 38.4 mM; $MgCl_2{\cdot}6H_2O$ 0.33 mM; $CaCl_2{\cdot}2H_2O$ 0.6 mM. The pH of the intestinal fluid was adjusted to 7.0 using 1 M HCl and monitored using a calibrated pH meter.

Briefly, the sample was subjected to a simulated gastric phase containing SGF, pepsin (2000 U/mL), and $CaCl_2{\cdot}2H_2O$ 0.15 mM, for 2 hours at 37°C. Subsequently, the gastric phase was mixed with SIF in a 1:1 volume ratio, which included $CaCl_2{\cdot}2H_2O$ at 0.6 mM. Pancreatin (trypsin activity 100 U/mL) and bile salts (10 mM) were then added to the intestinal phase. The solution was mixed for 2 hours at 37°C. Finally, the enzymes were inactivated by heating at 95°C for 10 minutes. In parallel, an INFOGEST blank (IB) sample was produced, following the same condition, replacing a corresponding volume of water instead of the sample. At the end of the intestinal digestive step, the enzymatic reactions were stopped at 95°C for 10 minutes. Then the digested GAF sample (iGAF) was subjected to a centrifugation step at 5500 RPM × 20 minutes at room temperature to remove the insoluble part. Subsequently, the recovered supernatant was subjected to a 3 kDa cut-off, for the complete removal of the inactivated enzymes, the retentate was discarded and the filtrate was collected and freeze-dried. From a starting material GAF of 5 g of, the freeze-drying yield was 3.6 g.

S.2.3. Amino acid (a.a.) analysis

The GAF and iGAF samples were diluted 1:1 with 0.2 N lithium citrate buffer pH 2.2. The solution was filtered on 0.2 μm membrane filter (Millipore, Milford MA, USA) and analysed by ion exchange chromatography using an amino acid analyser Biochrom 30+ (Erreci, Milan, Italy) as described by Hogenboom et al. (Hogenboom et al., 2017). The content of individual amino acids was calculated using five-level calibration curves.

S.2.4. Cell Culture

Caco-2 cells and STC-1 cells were routinely sub-cultured following a previously optimized protocol (2)and maintained at 37°C in a 90% air/10% CO_2 atmosphere in DMEM containing 25 mM of glucose, 3.7 g/L of NaHCO3, 4 mM of stable L-glutamine, 1% non-essential amino acids, 100 U/L of penicillin and 100 μg/L of streptomycin (complete medium), supplemented with 10% heat-inactivated FBS. Cells used in the experiments were between passage 5 and passage 20.For the co-culture, the STC- 1 and Caco-2 cells were cultured in a 1:5 ratio for 48 h before proceeding with treatments.

S.2.5. Caco-2 cells Differentiation

For Caco-2 cells differentiation, cells were seeded onto polycarbonate Transwell filters (12 mm diameter, 0.4 μm pore size; Corning Inc., Lowell, MA, USA) at a density of 3.5×10^5 cells/cm^2 in complete medium supplemented with 10% foetal bovine serum (FBS) in both apical (AP) and basolateral (BL) compartments for 2 days to promote the formation

of a confluent monolayer. From day 3 post-seeding, the culture medium was replaced with FBS-free medium in both compartments. Cells were maintained under these conditions for 18–21 days, with medium changes performed three times per week following a previously optimized protocol (3).

S.2.6. Caco-2 cells Monolayers Integrity Evaluation

The transepithelial electrical resistance (TEER) of differentiated Caco-2 cells was measured at 37 ◦C using the voltmeter apparatus Millicell (Millipore Co., Billerica, MA, USA), immediately before, after 15, 30, 60 and 120 minutes. Only filters showing TEER values similar to untreated control cells were considered for peptide transport analysis.

S.2.7. Amino acids uptake by intestinal monolayers

The iGAF intestinal absorption was assessed in transport buffer solution (137 mM NaCl, 5.36 mM KCl, 1.26 mM $CaCl_2$, and 1.1 mM $MgCl_2$, 5.5 mM glucose). The apical solutions were kept at pH 6.0 (buffered with 10 mM morpholinoethane sulfonic acid) and the basolateral (BL) solutions were kept at pH 7.4 (buffered with 10 mM N2hydroxyethylpiperazine-N4butanesulfonic acid) to replicate the pH conditions found in vivo in the small intestinal mucosa. Before the transport assay, cells were equilibrated in HBSS for 15 min at 37 °C. iGAF 0.3 mg/cm^2 was added to the apical compartment by dissolving it in transport solution (500 μL), while in the BL compartment was added the BL transport solution (700 μL). The apical solutions and the BL solutions collected at the end of the absorption experiment carried out for 1 h at 37°C, were analyzed by ion exchange chromatography using an amino acid analyser Biochrom 30+ (Erreci, Milan, Italy).

S.2.8. Cells treatments conditions

The experiments at cellular level on Caco-2 cells were designed to closely mimic the physiological conditions of intestinal absorption of the digested product, composed by amino acids. Specifically, since the recommended GAF daily dose is 5 tablets (5 g of product), and that the final yield after simulated digestion using the INFOGEST protocol is 3.6 g of dry product, it is assumed that this amount of digested material would theoretically interact with an absorptive intestinal surface. In particular, the surface area of the duodenal intestinal tract (A) was calculated according to Helander et al., following the previously described formula (4).

$$A = L \times D \times \pi \times LM$$

where L is the length of the duodenal tract (30 cm); D is the diameter of the duodenal tract (4 cm); LM is the surface amplification factor due to microvilli (6.5).

The duodenal intestinal absorbent area was calculated as approximately 2450 cm^2. To replicate *in situ* this *in vivo* sample-to-surface ratio, the amount of iGAF that makes in touch with cells was calculated based on the surface area of the culture wells. Therefore, as 3.6 g of iGAF come in contact with an intestinal absorbent area of 2450 cm^2, 3 mg of iGAF interacts with 2 cm^2 (0.32 cm^2 × 6.5 accounting for microvilli amplification) of *in vitro* absorbent surface area. iGAF was tested at a fixed surface dose of 10 mg/cm^2. In 96-well plates, where each well has a surface area of 0.32 cm^2, the cells were treated with approximately 3 mg of iGAF. For the treatments on STC-1 cells, due to their high sensitivity, we employed 0.3 and 1.5 mg/cm^2..

S.2.9. 3-(4,5-Dimethylthiazol-2-yl)-2,5-Diphenyltetrazolium Bromide (MTT) Assay

A total of 3 x 10^4 Caco-2 cells/well and 6 x 10^3 STC-1 cells/well were seeded in 96-well plates. Caco-2 cells were treated with GAF, iGAF and IB 10 mg/cm^2 or vehicle (H_2O) in

complete growth medium for 2 h, at 37 °C under a 5% CO_2 atmosphere, following the procedure previously reported (5). STC-1 cells were treated with iGAF up to 10 mg/cm^2. For the co-culture system, a total of 2.4 x 10^4 Caco- 2 cells and 6 x 10^3 STC-1 cells/well were seeded in 96-well plates and treated with iGAF and/or vehicle (H_2O) at 0.3 and 1.5 mg/cm^2, following the same conditions described above. In 96- well plates, being the surface area of 0.32 cm^2, the cells were treated with 10 mg/cm^2 of sample received 3 mg of sample.

S.2.10. Antidiabetic activity of GAF

S.2. 10.1. In vitro measurement of the DPP-IV inhibitory activity

The experiments were carried out in triplicate in a half volume 96 well solid plate (white) using conditions previously optimized (6). A total of 100.0 μL of each reaction was prepared in a microcentrifuge tube adding 70.0 μL of 1 × assay buffer [20mM Tris-HCl, pH 8.0, containing 100 mM NaCl, and 1 mM EDTA], 20.0 μL of GAF, iGAF, IB (at the final concentration of 1.0, 5.0, and 10.0 mg/mL), or sitagliptin at 1.0 μM (positive control) and 10.0 μL of purified human recombinant DPP-IV enzyme. Next, reagents were transferred in each well of the plate and each reaction was started by adding 100.0 μL of substrate solution (5mM H-Gly-Pro-AMC) and incubated at 37 °C for 30 min. Fluorescence signals were measured using the Synergy H1 fluorescent plate reader from Biotek (excitation/emission wavelength 360/465 nm).

S.2. 10.2. Evaluation of the Inhibitory Effect of iGAF on Cellular DPP-IV Activity

The experimental procedure was conducted following previous published methodology(7). A total of 3 x 10^4 Caco-2 cells/well were seeded in black 96-well plates with clear bottom. The second day after seeding, the spent medium was discarded and cells were treated with iGAF 10 mg/cm^2 or vehicle (C) in growth medium for 15 minutes at 37°C. Afterwards, treatments were removed and Caco-2 cells were washed once with 100 μL of PBS without Ca^{2+} and Mg^{2+}, before the addition to each well of 100 μL of Gly-Pro-AMC substrate at the concentration of 50.0 μM in PBS without Ca^{2+} and Mg^{2+}. Fluorescence signals deriving from the release of free AMC were measured using a Synergy H1 fluorescence microplate reader from BioTek (excitation/emission wavelength 350/465 nm respectively) every 1 min for up to 10 minutes.

S.2. 10.3. Evaluation of the GLP-1 stability and secretion at cellular level

STC-1 GLP-1 secretion was measured by an active GLP-1 ELISA kit (catalog no. EGLP-35K; Millipore, Watford, UK). In details, a total of 2.4 x 10^4 Caco-2 cells and 6 x 10^3 STC-1 cells/well or 6 x 10^3 STC-1 cells/well were seeded in 96-well plates. After 48h, cells were treated with Sitagliptin (1 μM), iGAF (final concentrations of 0.3 and 1.5 mg/cm^2), blank or vehicle (C) in growth medium for 1h. After the treatment, the supernatant was collected and centrifugated at 500×g, 4°C for 5 min and incubated in 96-well microplates coated with a monoclonal antibody for overnight (20 to 24 hours) at °4C. After washing the wells, the detection conjugated was added for 2h. The wells were washed, and then the substrate solution was added for 20 min. The reaction was stopped by a stop solution, and then the plate was read to with an excitation/emission wavelength of 355 nm/460 nm with a Synergy H1 microplate reader (Biotek Instruments, Winooski, VT, USA).

S.2. 11. Statical Analysis

Results were presented as the mean ± standard deviation (s.d.), and all measurements were carried out at least in triplicate. All the data sets were checked for normal distribution by D'Agostino and Pearson test. Since they are all normally disturbed with p-values < 0.05, we proceeded with statistical analyses with p-values < 0.05 deemed

significant. Dunnett's and Tukey's post-tests were conducted after statistical analyses using One- and Two-way ANOVA (Graphpad Prism 9, GraphPad Software, La Jolla, CA, USA).

S3. Supplementary results

S.3.1. IB *in vitro* DPP-IV inhibitory properties

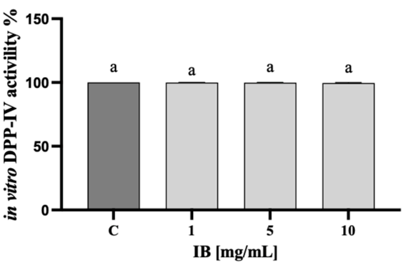

Figure S1. Effect of Infogest Blank (IB) on the *in vitro* DPP-IV activity. The data points represent the averages ± SD of 4 independent experiments performed in triplicate. All data sets were analyzed by one-way ANOVA followed by Tukey's post-hoc test. ns: not significant; C: control sample (H2O). Same lowercase letters indicate a no difference (p > 0.05) between different treatments.

S.3.2. Evaluation of Cells viability

S.3.2. 1. Evaluation of Caco-2 cells viability

Before proceeding to experiments on Caco-2, it was necessary to perform the MTT experiments for verifying that GAF, iGAF and IB did not impair the cellular vitality. The results (Figure S2) suggested that the samples were safe for intestinal cells treated with 10 mg/cm^2 (that corresponds to 3 mg in 0.32 cm^2 for 96-wells plate). The results clearly suggest that all the samples are able to preserve the Caco-2 cellular vitality, supporting its safety at intestinal level.

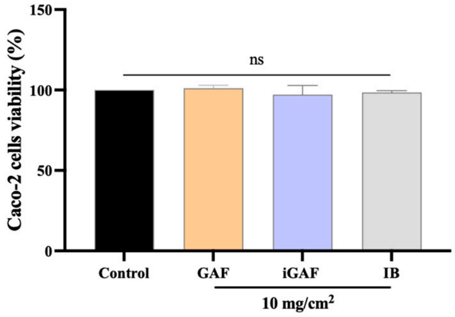

Figure S2. Evaluation of Gunaminoformula (GAF), INFOGEST Gunaminoformula (iGAF) and Infogest Blank (IB) effect on human intestinal Caco-2 viability. Data represent the mean ± s.d. of six determinations performed in triplicate. All data sets were analyzed by One-way ANOVA followed by Tukey's post-hoc test. ns: not significant; C: control, untreated cells; ns: not significant.

S.3.2.2. Evaluation of STC-1 cells viability

Before proceeding to experiments on STC-1, it was necessary to perform the MTT experiments for verifying that iGAF did not impair the cellular vitality. The results (Figure S3) suggested that the samples were safe for STC-1 cells when treated with 0.3 and 1.5 mg/cm^2.

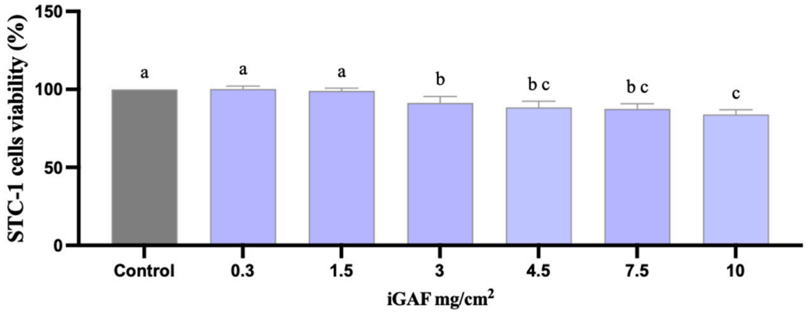

Figure S3. INFOGEST Gunaminoformula's (iGAF) citotoxicity evaluation on enteroendocrine STC-1 cells viability. Data represent the mean ± s.d. of six determinations performed in triplicate. All data sets were analyzed by One-way ANOVA followed by Tukey's post-hoc test. ns: not significant; C: control, untreated cells; ns: not significant. Different lowercase letters indicate a significant difference ($p < 0.05$) between different treatments.

S.3.2. Analysis of amino acids absorption by Caco-2 cells.

The results in Figure S4 shows the percentage of individual amino acids absorbed and transported to the basolateral (BL) compartment after treatment of the intestinal cells with iGAF (0.3 mg/cm²), in the Transwell system. The observed reduction in amino acid concentration in the initial solution, along with their appearance in the BL compartment, demonstrates active transport across the intestinal epithelium model. These results confirm that Caco-2 cells absorb and translocate amino acids, thereby modifying their concentration in the culture medium.

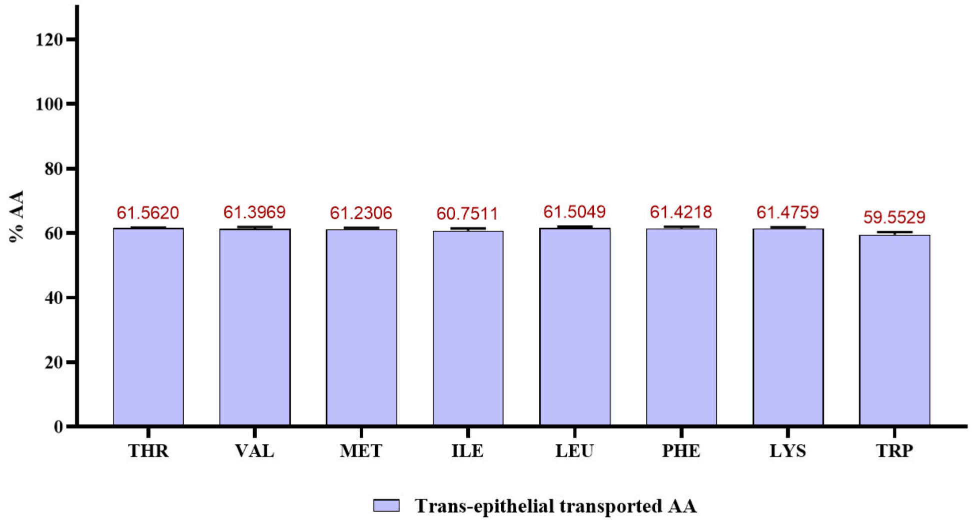

Figure S4. % of trans-epithelial transported amino acid after INFOGEST Gunaminoformula (iGAF) treatment. Cells were incubated with iGAF at 0.3 mg/cm² for 1h. The collected apical and BL solutions were analyzed by ion exchange chromatography using an amino acid analyser Biochrom 30+ (Erreci, Milan, Italy). The data points represent the averages of 3 independent experiments (technical and biological replicates). THR: Threonine; VAL: Valine; MET: Methionine; ILE: Isoleucine; LEU: Leucine; PHE: Phenylalanine; LYS: Lysine; TRP: Tryptophan.

References

1. Brodkorb A, Egger L, Alminger M, et al. INFOGEST static in vitro simulation of gastrointestinal food digestion. *Nature Protocols 2019 14:4*. 2019;14(4):991-1014. doi:10.1038/s41596-018-0119-1
2. Bartolomei M, Capriotti AL, Li Y, et al. Exploitation of Olive (Olea europaea L.) Seed Proteins as Upgraded Source of Bioactive Peptides with Multifunctional Properties: Focus on Antioxidant and Dipeptidyl-Dipeptidase—IV Inhibitory Activities, and Glucagon-like Peptide 1 Improved Modulation. *Antioxidants*. 2022;11(9). doi:10.3390/antiox11091730
3. Ferruzza S, Rossi C, Sambuy Y, Scarino ML. Serum-reduced and serum-free media for differentiation of Caco-2 cells. *ALTEX*. 2013;30(2):159-168. doi:10.14573/ALTEX.2013.2.159
4. Helander HF, Fändriks L. Surface area of the digestive tract - revisited. *Scand J Gastroenterol*. 2014;49(6):681-689. doi:10.3109/00365521.2014.898326
5. d'Adduzio L, Fanzaga M, Capriotti AL, et al. Ultrasonication coupled to enzymatic hydrolysis of soybean okara proteins for producing bioactive and bioavailable peptides. *Curr Res Food Sci*. 2024;9. doi:10.1016/J.CRFS.2024.100919
6. Bollati C, Xu R, Boschin G, et al. Integrated Evaluation of the Multifunctional DPP-IV and ACE Inhibitory Effect of Soybean and Pea Protein Hydrolysates. *Nutrients*. 2022;14(12):2379. doi:10.3390/NU14122379/S1
7. Lammi C, Bollati C, Ferruzza S, Ranaldi G, Sambuy Y, Arnoldi A. Soybean-and lupin-derived peptides inhibit DPP-IV activity on in situ human intestinal Caco-2 cells and ex vivo human serum. *Nutrients*. 2018;10(8):1-11. doi:10.3390/nu10081082
